# Untargeted metabolomics profiling in a mouse model of lung cancer treated with thermal ablation

**DOI:** 10.1080/21655979.2022.2065742

**Published:** 2022-04-28

**Authors:** Haoyue Pang, Kaiwen Hu, Fuyao Li, Hua Duan, Yu Chen, Yaqi Hu, Dan Wang, Min Jiang

**Affiliations:** a Graduate School, Beijing University of Chinese Medicine, Beijing, China; b Department of Oncology, Dongfang Hospital Beijing University of Chinese Medicine, Beijing, China; c Department of Hemooncology, Dongzhimen Hospital Beijing University of Chinese Medicine, Beijing, China

**Keywords:** Thermal ablation, cryoablation, hyperthermal ablation, metabolomics, lung cancer

## Abstract

Thermal ablation is widely used in the treatment of lung cancer and is beneficial for the overall survival of patients in clinic. However, there is barely a priority in which ablation system should be chosen under different periods of tumor progression in lung cancer. The present study investigated different modes of thermal ablation systems in mice with transplanted Lewis lung carcinoma tumors and their various biological effects in local regions using untargeted metabolomics. The results showed that thermal ablation could significantly suppress tumor growth and the differentially expressed metabolites of tumors after ablation relative to untreated tumors concentrated on organic compounds, organic acids and derivatives, nucleosides, nucleotides, and lipids. The upregulated metabolites indicated an inflammatory reaction in the ablation groups at an early stage after ablation. Steroid hormone and tryptophan metabolism, which are associated with immune responses, were modulated after both cryoablation and hyperthermal ablation. Characteristically, the results also indicated that cryoablation suppressed glucose oxidation and carbohydrate metabolism of tumor, while hyperthermal ablation suppressed lipid metabolism of tumor. In conclusion, thermal ablation could inhibit tumor growth under either freezing or heating modes with characteristic different biological effects on tumors.

## Graphical abstract

**Figure:**
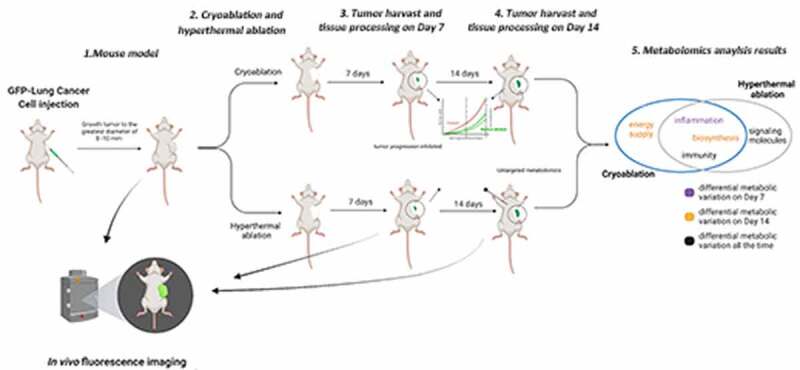

## Highlights

- Thermal ablation modulated carbohydrate,nucleosides, and lipids metabolism of lung cancer.
- Both cryoablation and hyperthermal ablation stimulated metabolites associated with inflammation and immune reaction.
- Glucose oxidation and carbohydrate metabo lism were significantly suppressed aftercryoablation
- lipid metabolism apparently altered after hyperthermal ablation.

## Introduction

Lung cancer is widely considered the most common cancer, with high mortality rates around the world. Almost 80% of lung cancer patients are diagnosed with non-small cell lung cancer (NSCLC), which has various clinical treatments. Local therapies, such as parenchymal sparing resection, radiation, and image-guided thermal ablation, have been recommended in the National Comprehensive Cancer Network (NCCN) Guidelines for the treatment of NSCLC, including both primary and metastatic cancers at early or advanced stages [1]. Currently, various ablation systems, including both freezing and heating, are widely applied in the treatment of lung cancer. The cryoablation could form ice crystals in the extracellular space, dehydrating tumor cells and causing damage to the vascular endothelial cells [2]. The thermal ablation at temperatures greater than 60°C would cause coagulative necrosis due to instantaneous protein denaturation [3]. The different biological effects caused by cold and heat damage to tumors arouse our research interest. The previous study has found that cryoablated tumors that remain within the target organ may potentially stimulate both innate and adaptive immunity [4]. Cryosurgery also facilitates anti-tumor immunoreactions via activation of anti-tumor immune cells, as well as increased secretion of proinflammatory cytokines [5]. A study on the efficacy of radiofrequency ablation in NSCLC showed that elevated levels of serum inflammatory factors, such as TNF-α, CCL-2, and CCL-4, were associated with early indicators of potential tumor relapse in NSCLC [6]. Therefore, our study focused on the biological effects of cryoablation and hyperthermal ablation and tried to provide a basis for their difference in the treatment effect.

Recently, metabolomics has been increasingly applied to the exploration of metabolic mechanisms and biomarker screening due to altered metabolism in lung cancer [7]. Nuclear magnetic resonance (NMR) and ultra-high performance liquid chromatography/tandem mass spectrometry (UPHLC-MS/MS) are used to characterize pre-treatment serum metabolites and lipid profiles in patients with NSCLC, which can help identify a high prognostic risk [8]. Moreover, some studies have also been conducted to predict the response to first-line chemotherapy and targeted drugs in patients with NSCLC by evaluating tumor-specific metabolic profiles [9,10].In this study, metabolite profiles would help us evaluate disease processes and physiological variations in NSCLC treated using different modes of ablation.

A new ablation system (HJY CHS 800001 Combined surgical system) was developed to integrate the advantages of cryoablation and hyperthermal ablation by decreasing the temperature to −196°C in freezing mode and increasing the temperature to 80°C in heating mode. With this technology, the study of the effects of freezing and heating ablation becomes more convenient and intuitive. In addition, previous experiments [11–13]and clinical trials [14] have proven the efficacy and safety of this system. Thus, our study evaluated metabolic profiles after performing the two modes of the combined ablation system to provide insight into the different effects of freezing and heating mechanisms.

## Materials and methods

Animal experiments were conducted after approval from the Institutional Animal Care and Use Committee (IACUC) of SPF Biotechnology Co.Ltd. The experimental protocol conformed to National Institutes of Health Guide for the Care and Use of Laboratory Animals.

### Mouse model

Thirty-six male C57BL/6 J mice (42–56 days old, 18–22 g), were purchased from SPF (Beijing) Biotechnology Co. Ltd (Beijing, China) and injected with the green fluorescent protein(GFP)-marked murine Lewis lung carcinoma (LLC-nb GFP) cells at 5 × 10^6^ cells per 200 µL PBS subcutaneously into the right subaxillary region (shaved with a sharp blade) to generate a lung cancer model [15].

### Cryoablation and hyperthermal ablation

When the tumors reached 8–10 mm at their greatest diameter 20 days after inoculation, mice were randomly assigned to one of three groups: model group (Model), cryoablation group (Cryo), hyperthermal ablation group (Therm), with 12 mice in each group. The mice were anesthetized with an intraperitoneal injection of pentobarbital sodium (10 mg/kg body weight) for surgery. The day of surgery was defined as ‘Day 0,’ and the surgery protocol of the ablation groups was as follows.

The surgery was operated using the HJY CHS 800001 Combined surgical system (Hygea Medical Technology Co., Beijing, China). The ablation probes, 1.66 mm in diameter, were inserted into the center of the exposed tumors with the maximum output power to form an ice ball until the temperature dropped to −140°C. When the ice ball reached the tumor boundaries without invading the skin tissue, freezing was stopped. The tumor was then thawed with anhydrous ethanol steam until the central tumor temperature reached 0°C, after which the probe was removed [16]. For hyperthermal ablation, the tumor was heated using anhydrous ethanol steam, the temperature of which reached as high as 80°C upon transition in the probe [14]. The probes were pulled out until the boundaries of the tumor were carbonized. Finally, the skin was sutured to cover the tumor after confirming no active bleeding. The mice recovered while maintained at 37–39°C and were returned to standard care with iodophor disinfectant applied to the wound once per day.

### Tumor measurements and harvest

The length and width of tumors were measured using a Vernier caliper every other day from Day 0 to Day 14. A small animal imaging system [17] was used to measure the tumor fluorescence intensity (FI) on Days 0, 6, and 12. Hair was removed from tumor tissues and mice were anesthetized with 1% isoflurane gas at a flow of 300 ml/min for 1 min before FI measurement. The mice were then placed on black paper in the imaging box to detect tumor cells. Tumor volumes were calculated using the following formula: tumor volume = length × width^2^ × 0.5. Three groups of mice were sacrificed to extract the tumor and lung tissue on Days 7 and 14 (n = 6). The tumors were weighed, rapidly frozen using liquid nitrogen, and stored at −80°C for metabolomic analysis. Lung tissue was fixed with 4% paraformaldehyde, embedded in paraffin, stained with hematoxylin and eosin (HE), and observed for pulmonary metastasis.

### Metabolite extraction

Tumor tissue (100 mg) was individually ground with liquid nitrogen and the homogenate was resuspended in prechilled 80% methanol and 0.1% formic acid using a well vortex. The samples were incubated on ice for 5 min and then centrifuged at 15,000 *g* at 4°C for 20 min. Some of the supernatants was diluted to a final concentration of 53% methanol using LC-MS grade water. The samples were subsequently transferred to a fresh Eppendorf tube and centrifuged at 15,000 *g* at 4°C for 20 min. Finally, the supernatant was injected into the liquid chromatography-tandem mass spectrometry (LC-MS/MS) system [18].

### UHPLC-MS/MS Analysis

UHPLC-MS/MS analyses [19,20] were performed using a Vanquish UHPLC system (ThermoFisher, Germany) coupled with an Orbitrap Q ExactiveTMHF-X mass spectrometer (ThermoFisher, Germany) under positive/negative polarity (Novogene Co., Ltd., Beijing, China). Samples were injected onto a Hypersil Gold column (100 × 2.1 mm, 1.9 μm) using a 17-min linear gradient at a flow rate of 0.2 mL/min. The eluent A was 0.1% formic acid (FA) in water for the positive polarity mode (ESI+) and eluent B was 5 mM ammonium acetate, pH 9.0 for the negative polarity mode (ESI−) with Methanol. The solvent gradient was 2% B, 1.5 min; 2–100% B, 12 min; 100% B, 14.0 min; 100–2% B, 14.1 min; 2% B, 17 min. Q Exactive^TM^ HF-X mass spectrometer was operated with a spray voltage of 3.2 kV, capillary temperature of 320°C, sheath gas flow rate of 40 arb, and aux gas flow rate of 10 arb. The raw data files generated by UPHLC-MS/MS analysis were processed using Compound Discoverer 3.1 (CD3.1, ThermoFisher) to perform peak alignment, peak picking, and quantitation for each metabolite. The main parameters were set as follows: retention time tolerance, 0.2 minutes; actual mass tolerance, 5 ppm; signal intensity tolerance, 30%; signal/noise ratio, 3; and minimum intensity,100, 000. After that, peak intensities were normalized to the total spectral intensity. The normalized data were used to predict the molecular formula based on the additive ions, molecular ion peaks, and fragment ions. The peaks were then matched with the mzCloud (http://www.mzcloud.org/), mzVault, and MassList databases to obtain accurate qualitative and quantitative results.

### Statistical analysis

SPSS 20.0 (International Business Machines Corp., USA) was used to analyze body weight, tumor volume, and fluorescence intensity data. Student’s t-test was used to compare continuous variables between two groups, and multiple comparisons were made using analysis of variance (ANOVA) with Dunn’s post-hoc multiple comparison test with an alpha level of 0.05. Statistical results are shown as mean ± standard deviation (SD) and were exported using GraphPad Prism 6 software. Principal components analysis (PCA) and Orthogonal partial least squares discriminant analysis (OPLS-DA) were performed using metaX, which is a flexible and comprehensive software for processing metabolomics data [21]. Volcano plots were used to filter metabolites of interest based on log_2_(fold change [FC]) and log_10_(p-value) of metabolites. These metabolites were normalized using z-scores for intensity areas and clustering using the Pheatmap package in R language after annotation using the HMDB database (http://hmdb.ca/metabolites) and LIPID Maps database (http://www.lipidmaps.org/). Receiver operating characteristic (ROC) curve analysis was performed to identify potential biomarkers of metabolites when the area under the ROC curve (AUC) > 0.99. The metabolic pathway enrichment for differentially expressed metabolites was assessed using the KEGG database (https://www.genome.jp/kegg/pathway.html); metabolic pathways were considered as enriched; when the p-value for metabolic pathway enrichment was <0.05, the metabolic pathway was considered significantly enriched.

## Results

The present study was to investigate the cryoablation and hyperthermal ablation in the treatment of lung cancer and their effects on the metabolism of tumors. Both cryoablation and hyperthermal ablation were verified to maintain tumor suppression for a long time after surgery. The untargeted metabolomics analysis was carried out on the Model, Cryo, and Therm groups at different time points. The differentially expressed metabolites included organic compounds, organic acids and derivatives, nucleosides, nucleotides, and lipids in tumors after thermal ablation. Glucose oxidation and mediate carbohydrate metabolism were significantly suppressed after cryoablation, while lipid metabolism apparently altered after hyperthermal ablation.

### Tumor growth and metastasis

**Figure 1.**
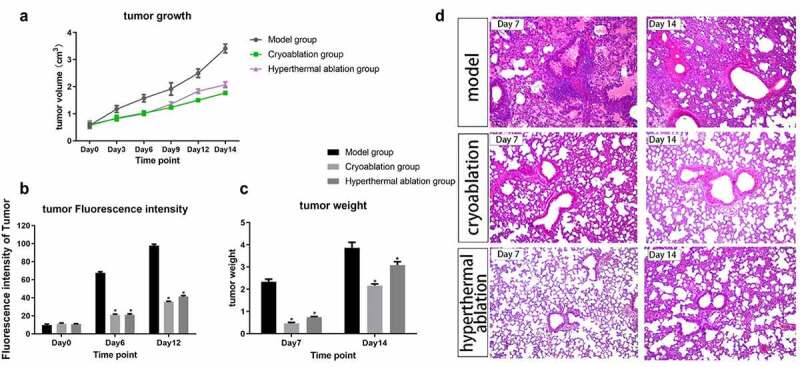
Dynamic changes in tumor volume (a), fluorescence intensity (b), and tumor weight (c) after intervention from Day 0 to Day 14. *P-value < 0.05 compared with model group. The graphs plotted the mean with SD error bars. (d) Histological review of lung tissues (×400) in the Model, Cryo, and Therm groups on Day 7 and Day 14.

The tumor volumes and FI of the three groups were comparable at baseline, and the ablations significantly inhibited tumor growth relative to that in the model group (Supplementary Table 1 and Figure 1a). In the ablation groups, the tumor volumes increased similarly during the first 7 days. The growth trend in the Therm group was sharper than that in the Cryo group from Day 8 to Day 14. In addition, the value of tumor FI in the ablation groups was much lower than that in the model group on Days 6 and 12 (Figure 1b). The tumor weight was measured on Days 7 and 14. The average weight in the Cryo and Therm groups was significantly different from that in the model group (P < 0.05); however, it was no difference between the Cryo and Therm groups (Figure 1c). The histological review showed that the lung tissues of the untreated mice were widely invaded by tumor cells, while more normal lung tissues were preserved in the ablation groups (Figure 1d). These results suggest that the growth and metastasis of tumors became much slower after ablation compared with that of untreated tumors.

### Metabolite screening after ablation

**Figure 2.**
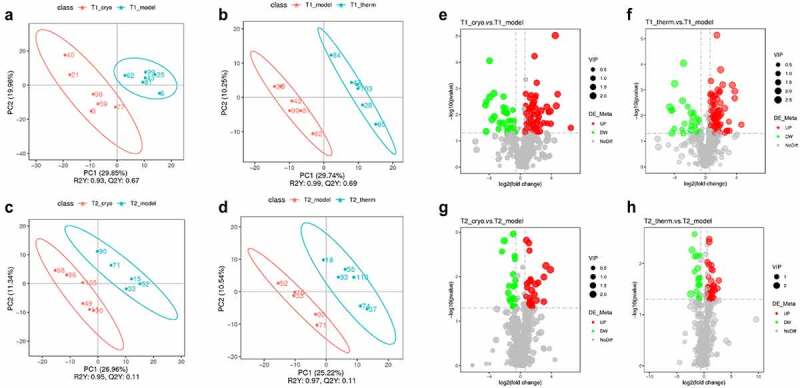
Differentially expressed metabolite screening results after the intervention. OPLS-DA score plots of Model vs. Cryo (a) and Model vs. Therm (b) on Day 7, and Model vs. Cryo (c) and Model vs. Therm (d) on Day 14 in ESI− mode. Volcano plots of Model vs. Cryo © and Model vs. Therm (f) on Day 7, and Model vs. Cryo (g) and Model vs. Therm (h) on Day 14 in ESI− mode. Each colorful point represents a differentially expressed metabolite of tumor tissue in the volcano plot. The red points represent up-regulated metabolites and the green points represent down-regulated metabolites.

The quantitative results of metabolites are shown in both ESI+ and ESI− modes, and quality control (QC) samples were analyzed using Pearson’s correlation coefficient [22] and PCA to evaluate their stability and accuracy. The results displayed in Supplementary Fig. 1 indicate that the instrument was stable and the data were reliable. The differentially expressed metabolites were identified following OPLS-DA analysis (Figure 2a–d and Supplementary Fig. 2A–D) between model and ablation groups referred to variable importance (VIP) in the significance with VIP > 1.0, FC > 1.5 or FC < 0.667, and P-value < 0.05, which are represented visually in volcano plots. On Day 7, there were 334 (Model vs. Cryo) and 161 (Model vs. Therm) differentially expressed metabolites in the ESI+ mode (Supplementary Fig. 2E & 2 F), while there were 186 and 100 metabolites, respectively, in the ESI− mode (Figure 2e & 2f). The number of differentially expressed metabolites identified following the Cryo and Therm groups relative to the model group were 230 and 102, and 118 and 55 in the ESI+ mode (Supplementary Fig. 2 G & 2 H) and ESI− mode (Figure 2g & 2h), respectively, on Day 14. The results suggest that both cryoablation and hyperthermal ablation could significantly change tumor metabolism; however, the residual effects weaken over time.

### Analysis of identified differentially expressed metabolites

**Figure 3.**
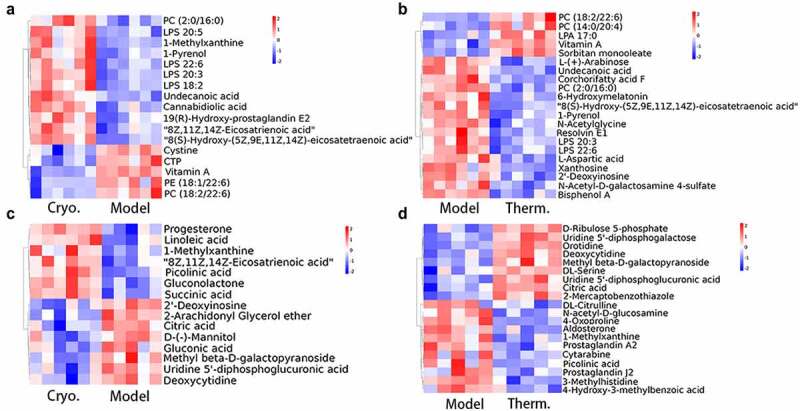
Identified differentially expressed metabolite clustering and enrichment in metabolic pathways. Heatmaps of differentially expressed metabolites clustering for Cryo vs. Model group (a), and Therm vs. Model group (b) on Day 7, and Cryo vs. Model group (c) and Therm vs. Model group (d) on Day 14 in ESI− mode.

**Figure 4.**
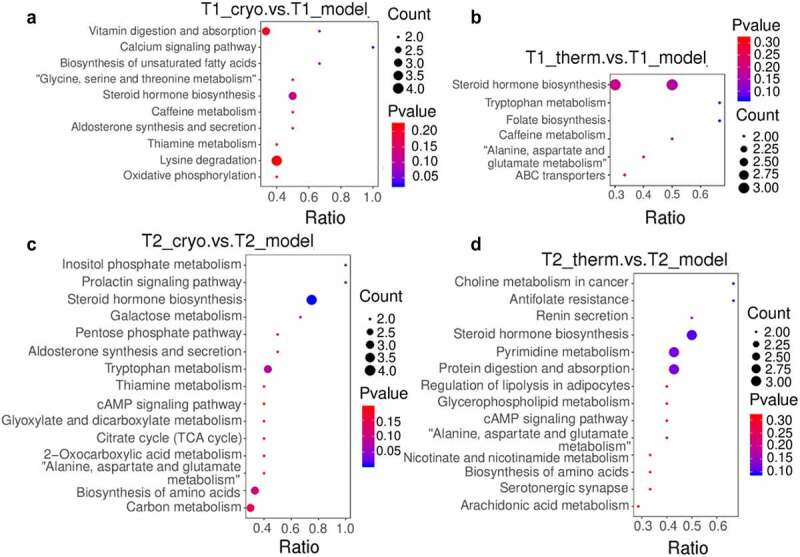
Bubble chart of KEGG pathway enrichment from differentially expressed metabolites for Cryo vs. Model group (a) and Therm vs. Model group (b) on Day 7, and Cryo vs. Model group (c) and Therm vs. Model group (d) on Day 14 in ESI− mode.

To clarify the categories and biological functions of the identified metabolites, the metabolites were annotated using the HMDB database and LIPID Maps database. The HMDB annotation totally identified 109 and 201 metabolites in the Cryo and Therm groups, respectively, that were differentially expressed with respect to the model group (Supplementary Fig. 3A & B). As shown in the HMDB annotation, there were a large number of lipids and lipid-like molecules among the identified differentially expressed metabolites; therefore, the metabolites were subjected to lipid annotation (Supplementary Fig. 3C & D). Fatty acids were upregulated while prenol lipids and hydroxyindoles were downregulated in both ablation groups on Day 7. The distinct variation in the Cryo group comprised upregulated metabolites, including glycerophospholipids and organic nitrogen compounds, as well as downregulated metabolites, including organooxygen compounds and some other lipid molecules. In addition, the expression of organooxygen compounds and urine nucleosides increased in the Therm group (Figure 3 a & b and Supplementary Fig. 4A & B). On Day 14, the steroids and steroid derivatives, as well as fatty acids, were overexpressed in both ablation groups. After cryoablation, the tumor demonstrated upregulation of metabolites such as prenol lipids and some organic compounds, as well as downregulation of metabolites such as pyrimidine nucleotides and organooxygen compounds (Figure 3c and Supplementary Fig. 4C). The expression of nucleosides, nucleotides, and analogs was decreased in the Therm group (Figure 3d and Supplementary Fig. 4D). In addition, carboxylic acids and derivatives appeared to demonstrate variations in both groups over time. Then, the analysis of KEGG pathway were performed between the model and ablation groups at different time points. Ten metabolic pathways were significantly enriched in the Cryo group, and six significant metabolic pathways were selected from the Therm group on Day 7 (Figure 4 a & b). On Day 14, differentially expressed metabolites were enriched in 15 metabolic pathways in the Cryo group and 14 metabolic pathways in the Therm group (Figure 4c & D). The results suggest that different metabolisms were modulated in tumors after disparate ablation approaches, and variable metabolic alterations occurred over time.

### Potential biomarkers for different modes of thermal ablation

**Figure 5.**
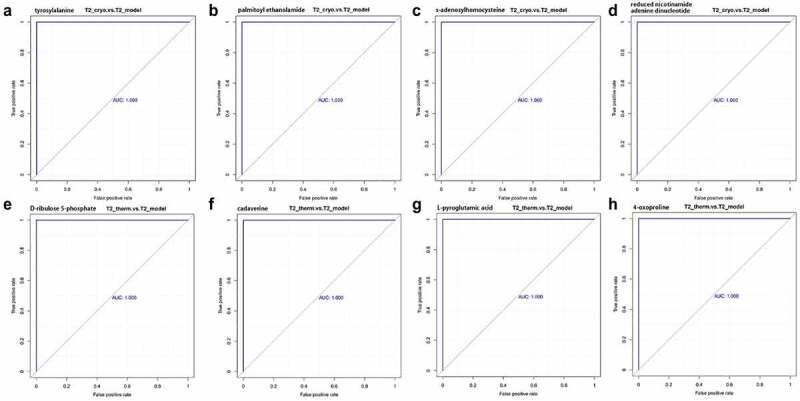
The AUC of ROC for potential biomarkers in Cryo and Therm groups. Tyrosylalanine (a), palmitoyl ethanolamide (b), s-adenosylhomocysteine(c), and reduced nicotinamide adenine dinucleotide (d) by Cryo vs. Model group. D-ribulose 5-phosphate (e), cadaverine (f), L-pyroglutamic acid (g), and 4-oxoproline (h) by Therm vs. Model group.

In order to find out potential biomarkers from differentially expressed metabolites in the ablated tumors, we operated ROC analysis based on the co-expression of metabolites in the Cryo and Therm groups on both Day 7 and Day 14. In the Cryo group, a total of 26 metabolites were co-expressed on different time points with four identified biomarkers including tyrosylalanine, palmitoyl ethanolamide, s-adenosylhomocysteine, and reduced nicotinamide adenine dinucleotide. Four metabolic biomarkers in the Therm group were selected from 25 co-expressed metabolites of different time points, which were D-ribulose 5-phosphate, cadaverine, L-pyroglutamic acid, and 4-oxoproline (Figure 5).

## Discussion

Thermal ablation offers NSCLC patients an effective and safe alternative therapy in clinic with preserved quality of life and prolonged life. It was reported that 40 patients with a total of 60 multiple lung metastases treated by 48 cryoablation sessions achieved a local tumor control rate of 96.6% and 94.2% at 6 and 12 months with a one-year overall survival rate of 97.5% [23]. A retrospective study of 12 patients who received thermal ablation (radiofrequency ablation or microwave ablation) for recurrent NSCLC after radiotherapy reported effective local tumor control with minimal recurrent morbidity [24]. In our study, the experimental results verify that thermal ablation inhibits tumor growth and invasion in – xenograft mice with lung cancer.

To further explore the variable effects of different ablations in freezing and heating modes, we investigated the metabolomic profiles of tumors at different time points after surgery. Significant changes occurred in tumor metabolism after ablation but these alterations reduced over time, which matched the growth trend of the ablated tumor. The analysis of identified metabolites indicated that the expressions of fatty acyls, glycerophospholipids, and prenol lipids were altered in both ablation groups (Figure 3). For instance, overexpressed fatty acyls and glycerophospholipids, such as eicosanoids, lysophosphatidylserine, and phosphatidyl choline, participate in cell signaling of inflammation and immune responses [25,26]. Downregulated metabolites, such as vitamin A [27] (prenol lipids), N-acetylsphingosine, N-acetylserotonin, and O-arachidonoyl ethanolamine (fatty acyls), could increase the survival likelihood of tumor, proliferation of cancer cells, and immune suppression [28]. The enriched pathways in both ablation groups, including steroid hormone biosynthesis and tryptophan metabolism, also contributed to tumor progression and immune evasion (Figure 4). Typical steroid hormones include corticosteroids and sex steroids. Glucocorticoids, such as cortisol, control cell metabolism and immune cell function regulating internal inflammation and immunity [29]. Sex steroids have been reported to play a positive role in the survival of patients with NSCLC by inducing less tumor cell proliferation [30]. Tryptophan metabolism can induce Treg cells differentiation and blunt antitumor T cells responses in the tumor microenvironment of glioblastoma [31]. These results suggest that thermal ablation may induce anti-inflammatory and immune reactions in tumor tissues.

Amino acid metabolism was another major alteration in both ablation groups, and there was a greater diversity in the Cryo group. The results showed that pyrimidine and purine were downregulated after cryoablation, while nucleosides, nucleotides, and analogs were the main downregulated metabolites after hyperthermal ablation over time (Figure 3). The metabolism of alanine, aspartate, and glutamate, which participate in the oxidation-reduction reaction of the glycolysis and tricarboxylic acid cycle (TCA cycle), was enriched in both ablation groups [32]. Other metabolic pathways, including glycine, serine, and threonine metabolism as well as lysine degradation, were enriched in the Cryo group (Figure 4). Glycine is synthesized from serine and threonine that can mediate metabolic disorders in cancer [33]. In contrast, lysine degradation is mainly involved in protein digestion and absorption, so the process of amino acid absorption and use in cancer cells might also be affected. To sum up, both modes of thermal ablation could cause tumor inhibition by altering amino acid metabolism, and cryoablation could induce more metabolite variations.

Furthermore, several altered metabolites identified in the Cryo group were associated with energy metabolism. Metabolic reprogramming is regarded as a core hallmark of cancer that provides sufficient energy for anabolic growth by increasing aerobic glycolysis and glutaminolysis over the TCA cycle [34]. The downregulated organooxygen compounds, including D-(-)-mannitol and gluconic acid generated from glucose oxidation, indicate a reduction in energy supply for cancer cells [35,36]. We also identified many pathways related to carbohydrate metabolism in the Cryo group (Figure 4). The substrates of carbohydrate metabolism provide the essential saccharides and energy for tumor growth. It seems that cryoablation could also modulate the energy metabolism in tumors.

Some identified differentially expressed metabolites of lipid metabolism serve as signaling molecules or secondary messengers in tumor progression. The upregulation of steroids and steroid derivatives, which act as enzyme inhibitors and cytotoxic drugs in cancer treatment [37], was found to have therapeutic potential. Steroids, such as prostaglandin A2 (PGA2) and prostaglandin J2 (PGJ20), were overexpressed after hyperthermal ablation. Some researchers have reported that PGA2 might induce apoptosis in cancer cells via activation of tumor suppressor protein 53, which is one of the most crucial tumor suppressor genes [38]. The A and J series of prostaglandins are both characterized by reactive alpha, beta-unsaturated carbonyl compounds, which might be critical for the control of DNA-binding activity [39]. The enriched lipid metabolism in the hyperthermal ablation group also included glycerophospholipid metabolism, regulation of lipolysis in adipocytes, and the closely related choline metabolism in cancer, all of which are related to signaling factors (Figure 4). These metabolic pathways participate in fatty acid metabolism and oncogenic signaling, which sustain tumor growth, invasion, and metastasis [40,41]. The previous study showed that transcription factor nuclear receptor related to the regulation of lipid metabolism was up-regulated in the residual hepatoblastoma that received incomplete hyperthermal ablation [42]. The regulation of lipid metabolism also occurred in lung cancer after hyperthermal ablation in our research. As a result, hyperthermal ablation tends to modulate lipid molecules involving in signaling transport in tumors.

Taking all these together, the differentially expressed metabolites and metabolic pathways associated with inflammation and immune responses were identified in both ablation groups and were more pronounced at the early stage after ablation. On the one hand, cryoablation had a greater impact on carbohydrate and amino acid metabolism, which contributes to protein digestion and absorption as well as energy metabolism. On the other hand, lipid metabolism varied extensively in tumors after hyperthermal ablation, which may disrupt the signal transduction cascade in cancer cells. Interestingly, the inflammatory reaction and immune response are probably due to tumor tissue necrosis after ablation, but the individual effects of two modes of ablations were first discovered. The AUC of ROC analysis was 1.00 for potential biomarkers, showing high predictive accuracy on different biological effects of both cryoablation and hyperthermal ablation. The results suggested that four metabolic biomarkers of each ablation could represent their mechanism in the treatment of lung cancer. However, additional verification is required to clarify the underlying molecular mechanisms. This finding may promote a more accurate application and therapeutic combination of thermal ablation in lung cancer treatment according to characteristics of different ablations.

## Conclusion

Thermal ablation could suppress tumor growth in both cold and heat mode, affecting different biological processes on tumor metabolism. The metabolites related to immune response was altered in both cryoablation and hyperthermal ablation group. And cryoablation also modulated tumor energy metabolism while hyperthermal ablation had a greater effect on lipid metabolism that functioned as signal molecules and metabolic materials of the tumor. Our study explored the differential effects of cryoablation and hyperthermal ablation in lung cancer treatment to provide a basis for the application of various ablation systems. The mechanical correlation between different forms of thermal ablation and clinical efficacy requires further study.

## Supplementary Material

Supplemental Material
