# Supplementary material for: Untargeted metabolomics profiling in a mouse model of lung cancer treated with thermal ablation
Source: Bioengineered. 2022 Apr 28;13(4):11258–68. doi: 10.1080/21655979.2022.2065742 (PMC9208470; doi:10.1080/21655979.2022.2065742)
Supplement: Supplemental Material [file KBIE_A_2065742_SM1189.docx]

Table 1. Dynamic changes of the tumor volumes and Fluorescence intensity after the intervention

| Time point | Model group ^a^ | | Cryoablation group ^a^ | | Hyperthermal ablation group ^a^ | |
| --- | --- | --- | --- | --- | --- | --- |
|  | Tumor volume ^b^ | Fluorescence intensity | Tumor volume ^b^ | Fluorescence intensity | Tumor volume ^b^ | Fluorescence intensity |
| Day 0 | 0.5713± 0.0925 | 9.73×10^9^±1.15 | 0.5697±0.1054 | 11.05×10^9^±1.00 | 0.6113±0.1262 | 10.82×10^9^±0.46 |
| Day 3 | 1.1710 ±0.1278 | \ | 0.8371±0.1000 | \ | 0.8144±0.0068 | \ |
| Day 6 | 1.5700±0.1391 | 67.56×10^9^±1.16 | 1.0206±0.0816 | 21.01×10^9^±0.86* | 0.9986±0.0617 | 21.28×10^9^±0.88* |
| Day 9 | 1.9143±0.2320 | \ | 1.2330±0.0734 | \ | 1353.51±0.0805 | \ |
| Day 12 | 2.4956±0.1605 | 97.56×10^9^±1.73 | 1.5032±0.0678 | 35.70×10^9^±0.36* | 1833.14±0.0798 | 41.44×10^9^±1.00* |
| Day 14 | 3.412±0.1623 | \ | 1.7638±0.0719 | \ | 2072.52±0.1059 | \ |

^a^ Results were displayed as mean ± SD. ^b^ The unit is mm^3^.

^*^ Compared with model group, P-value < 0.05


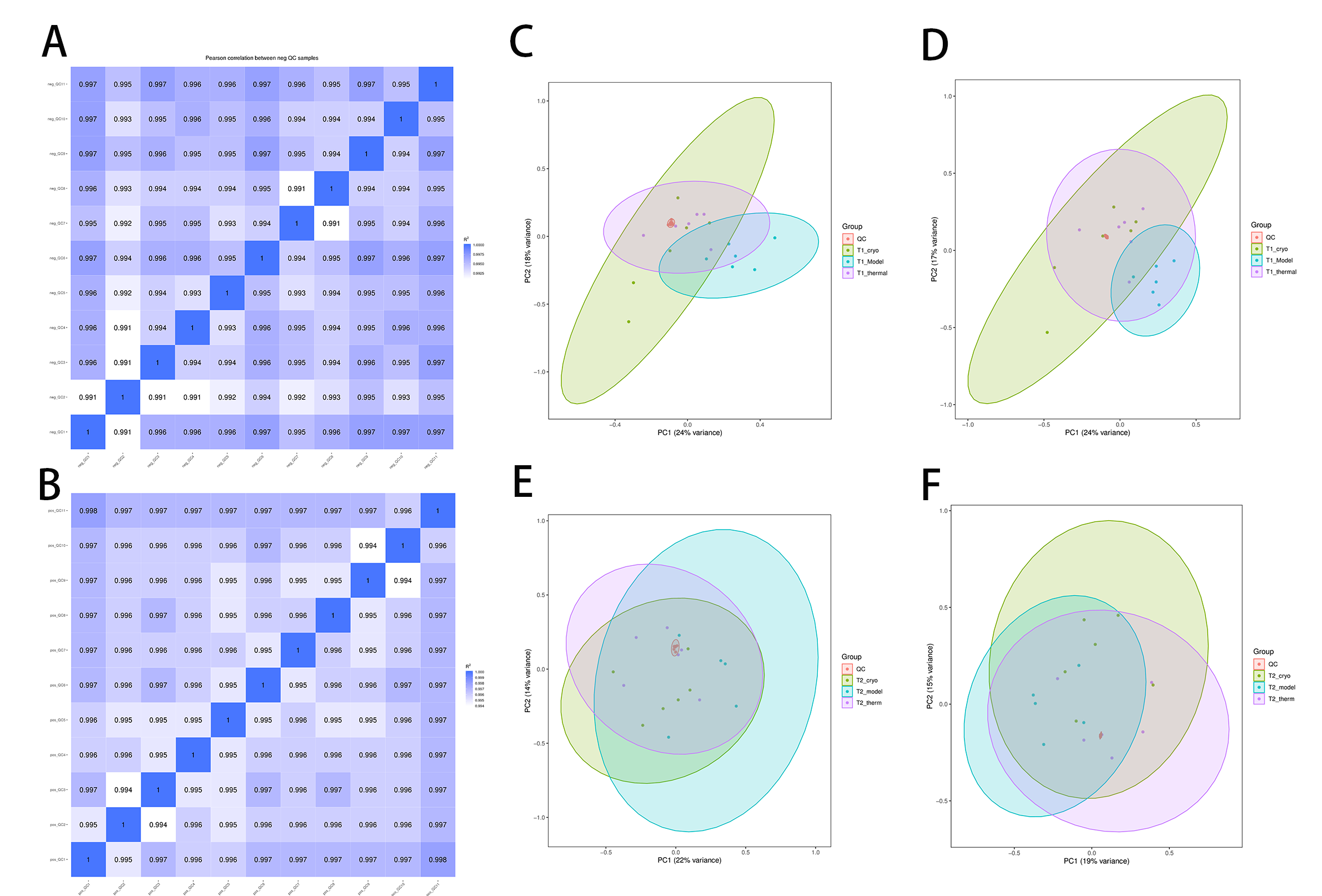


**Supplementary Fig. 1** QC samples analysis. The pearson correlation coefficient in the ESI+ (A) and ESI− (B) mode. Principal component analysis (PCA) score scatter plots in ESI+ (C) and ESI− (D) mode on Day 7 and ESI+ (E) and ESI− (F) mode on Day 14.


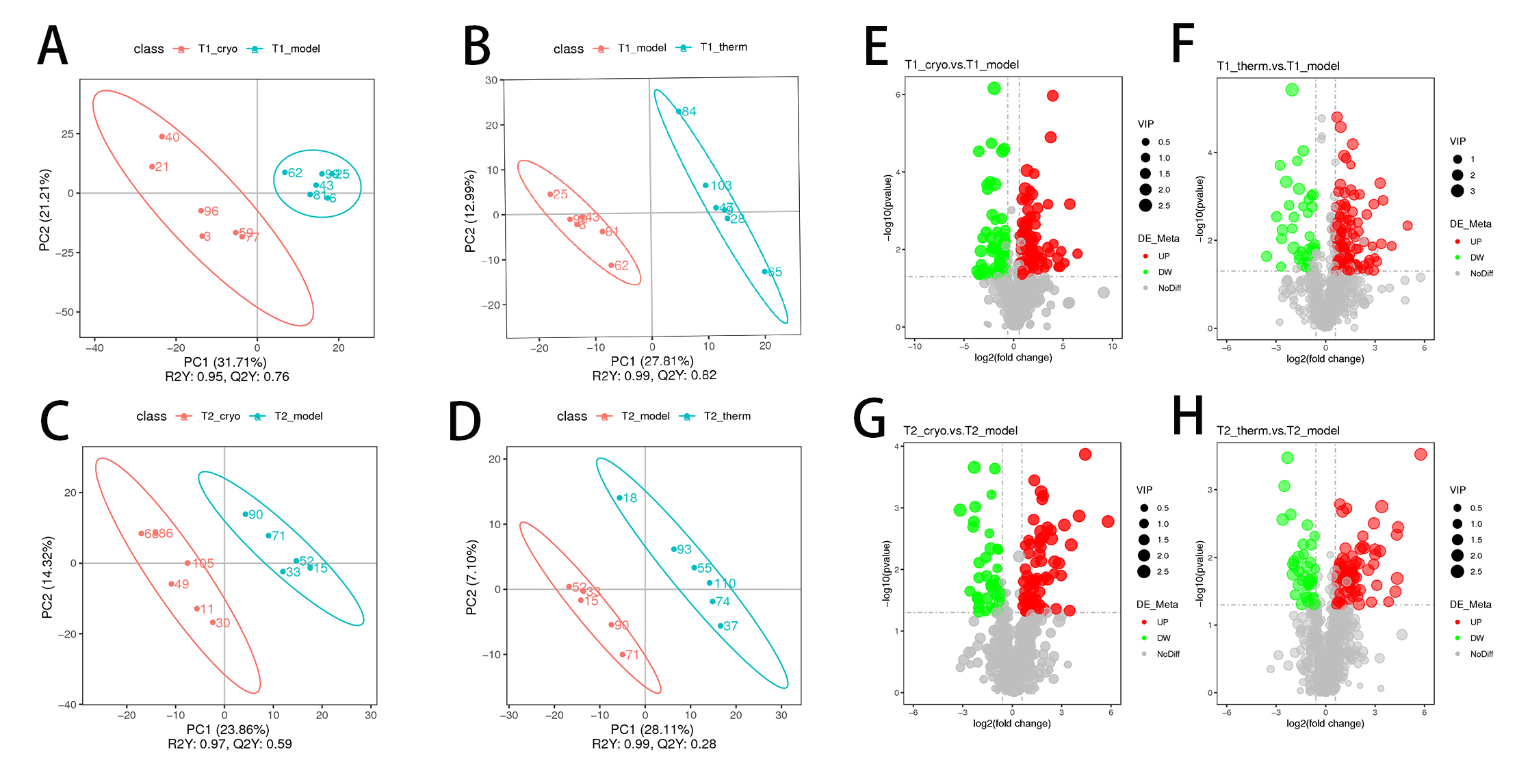


**Supplementary Fig.2** Differential metabolites screening after intervention. Orthogonal partial least squares-discriminant analysis (OPLS-DA) score plots of Model vs. Cryo (A) and Model vs. Therm (B) on Day 7, and Model vs. Cryo (C) and Model vs. Therm (D) on Day 14 in ESI+ mode. Volcano plots of Model vs. Cryo (E) and Model vs. Therm (F) on Day 7, and Model vs. Cryo (G) and Model vs. Therm (H) on Day 14 in ESI+ mode.


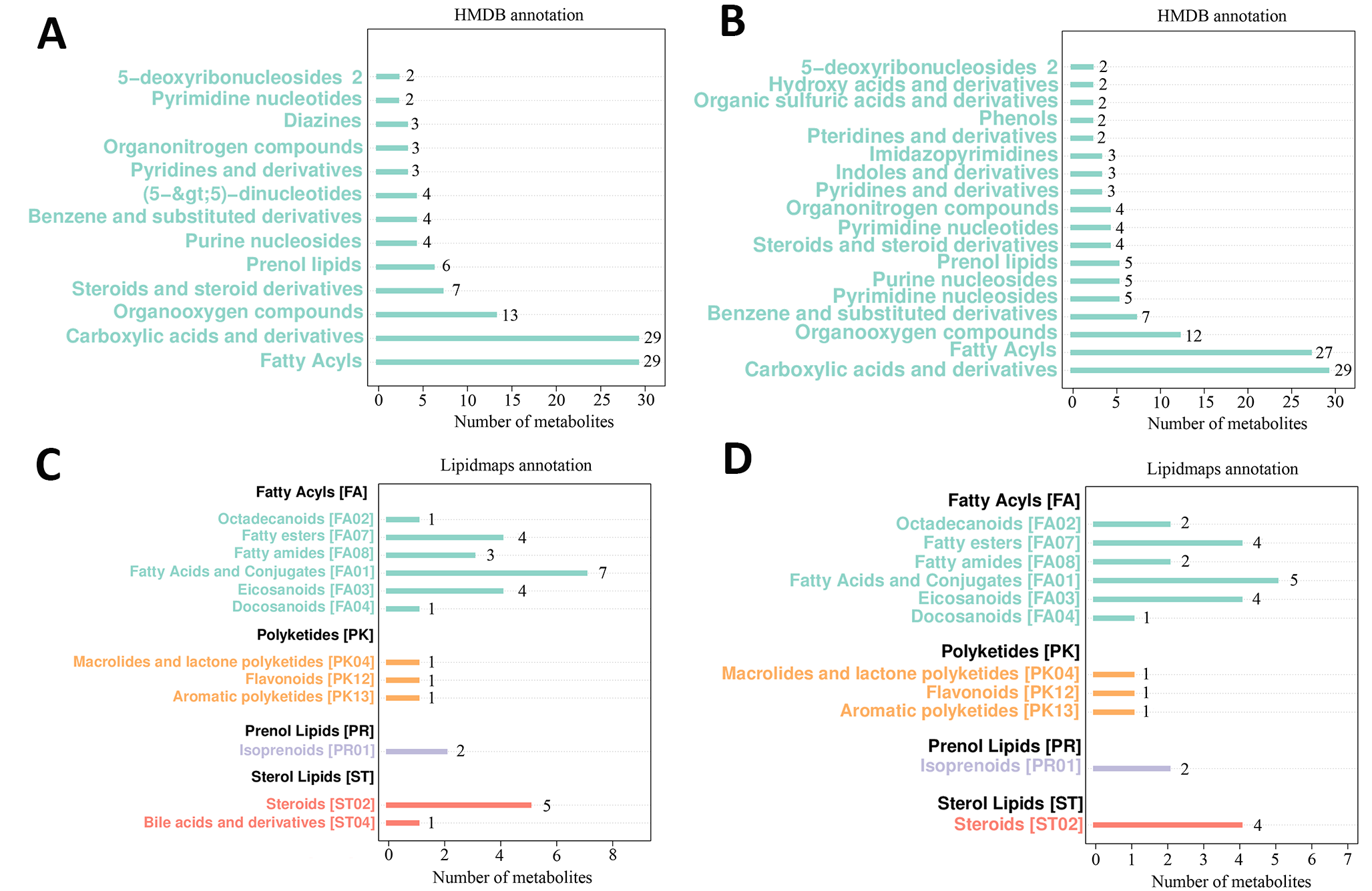


**Supplementary Fig.3** Differential metabolites annotation after intervention. The HMDB annotation of Cryo vs. Model group (A), model Therm vs. Model group (B) and Lipidmaps annotation of Cryo vs. Model group (C), Therm vs. Model group (D).


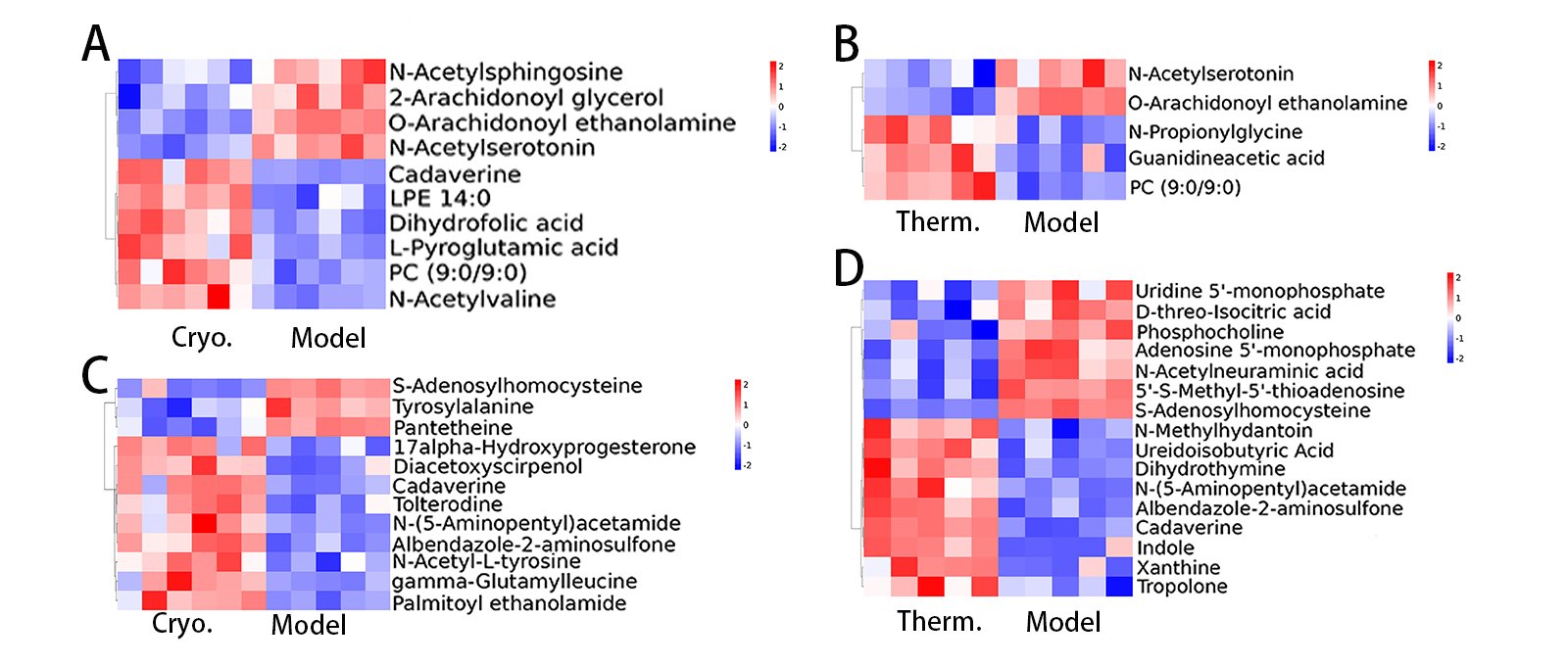


**Supplementary Fig.4** Identified differential metabolites clustering and enrich in metabolic pathways. Heat maps of differentially expressed metabolites clustering for Cryo vs. Model group (A), and Therm vs. Model group (B) on Day 7, and Cryo vs. Model group (C) and Therm vs. Model group (D) on Day 14 in ESI+ mode.
